# The stuff and nonsense of open data in government

**DOI:** 10.1038/sdata.2017.131

**Published:** 2017-09-19

**Authors:** Ian L. Boyd

**Affiliations:** 1 Department for Environment, Food and Rural Affairs, 17 Smith SquareRA, London SW1P 3JR, UK; 2 University of St Andrews, College Gate, North Street, St Andrews KY16 9LB, Scotland

**Keywords:** Policy, Research data

## Comment

When I think of data I think of binary or hexadecimal numbers. This betrays something of my background, but it was a surprise to me when in Defra, the UK Department of State with responsibility for food and the environment, we started to talk about data and I found that other people saw data very differently. Everybody had different preconceptions about data. Some seemed to be very confused. It had become trendy to talk about data, but few people appeared to think about data.

This can cause problems when it comes to using data to inform decisions, and building consensus within a large corporate body for its people to work towards a common goal. Unless everyone operates to similar definitions and uses common language, then this could result in a lot of nugatory work. Across government there may be just about as many definitions of data as there are people. Sometimes even data specialists seem confused as to what we mean when we say ‘data’.

This diffuse definition is a particular problem because not everybody understands how data contributes to knowledge and evidence. There is a unidirectional flow of logic, from data to information to knowledge to evidence. Evidence is what decision-makers really seek, but data are not evidence until they have been through an interpretive sieve, and evidence is definitely not just data as some people sometimes seem to think. Data helps decision-makers to get the evidence they need, but data are not information unless one can detect structures or patterns in them, and information is not knowledge unless those patterns have been verified by statistical analysis and their implications understood. Knowledge is not evidence unless it is used to address specific questions in a given context.

If I turn my mind to information theory in the context of natural ecosystems (the area of my research and expertise) then I could just about get away with describing those systems as natural repositories of data. For example, DNA stores the data template for the development and operation of organisms. Synthetic DNA may even be a potential chemical store for recoverable data. Scaling up, whole organisms or whole natural systems could be seen as data stores.

To illustrate this, in an earlier part of my career, I used the growth patterns in the teeth of animals to detect climatic variation; others use tree rings for the same purpose. The teeth and the trees are data stores. Extracting those data can be very laborious and verifying the interpretation so that it becomes ‘information’ can be difficult, but these may be the only ways of gaining knowledge about historical climate variability in times before there were any modern instruments. Fundamentally there is little difference between these kinds of data and the data in a digital image except that the extraction algorithm for image files is better known and works quickly, and the information in the image is easier to interpret and turn into knowledge. But a problem that the teeth and tree ring example illustrates is that just about everything, everywhere could be defined as data!

Unless we can settle on a clear and consistent definition then I think ‘data’ will present a bundle of problems when it comes to how big organisations respond to the challenge of creating operational solutions to the storage, management and applications of the kind of stuff we tend to call data. The definition seems to be infinitely extensible. The Oxford English Dictionary defines data as ‘facts and statistics collected together for reference or analysis’. By this definition, the OED is itself data. If we accept this definition, it implies that the facts and statistics need to have purpose and I think this is the key. I suggest that ‘data’ do not become data unless they are used for a defined purpose. Otherwise data are just ‘stuff’.

I have good reasons for suggesting this. First, storing vast amounts of data without a purpose will very quickly become unachievable. For example, if it continues that 90% of data in current existence has been collected in the past two years then this will quickly exceed storage capacity. We will have to make choices about what to keep and what to throw away. The very act of choosing forces a judgement to be made about what the purpose of the data might be. Second, if data have purpose, they become possible to regulate more intelligently based on that purpose, rather than trying to regulate the existence of the data itself.

I’m a strong supporter of making non-personal data open data and Defra has recently made much of its data open. However, in reality the more than 12,000 data sets now published by Defra as open data are just the start of what it is trying to achieve. It’s actually more of a statement about open government—Defra is happy to open its books so that others can use the stuff that flows around inside Defra—than about data per se. Defra wants people to use this stuff, but more important is that when people do use the stuff released by Defra they should be doing this within a clear framework of regulation that draws red lines beyond which unacceptable use lies.

So, this emphasises the real problem. Once this stuff is open then anybody can access it from all sorts of different jurisdictions, many of which won’t apply the same levels of scrutiny as we might in the UK. This has recently been illustrated to me in the field of earth observation. Here, there are increasing numbers of satellites passing overhead photographing the ground. If they photograph our garden on a regular basis, how would we feel about the receivers of the data in these photographs keeping an eye on us? If you’re a farmer it can say what crops you are growing and what commercial choices you are making. At some resolutions it might allow people to track your movements automatically. Would you tolerate your next-door neighbour poking a camera over the fence and snapping a picture of your garden on a sub-daily basis and then posting the pictures on the web?

The public are rightly concerned about the use of big data analysis by private companies to gain market edge and also by governments to use data to push their agendas. There is a sense that those who control the information from data potentially control people’s lives. Artificial intelligence is often seen as something being applied to replace, and improve upon, common human activities like playing a complex game or in the creation of intelligent robots to replace human labourers. But when artificial intelligence is applied to doing things that individual humans cannot do, like pattern recognition across massive flows of ‘stuff’, the outcome could be very different to any past experience.

Government is mostly involved in creating or implementing policies concerning how people live their lives. Making government data open could be seen as the equivalent of open government itself because data are increasingly the ‘stuff’ of government. But there are moral and ethical issues concerning the data owned by government and there is an issue of trust about how government handles data. The more obvious issues concerning data that are tagged specifically with the identity of an individual are probably well covered, but we all know that this is not sufficient. We need to be aware of the pitfalls about opening government without appropriate assurances around the ethical and moral use of the data it holds.

It would be wrong, however, to always see the risks presented by open data and not also see the benefits. Like all innovations it is important to design the application of the innovation to maximise benefits and minimise risks. The intelligent use of data could revolutionise government and place a lot more control in the hands of individuals by, for example, ensuring that everybody can have instant access to all the information that government holds about them and can make their own decisions about who should be allowed to see that information and what uses can be made of it. There is a big drive in government towards this kind of model of data control. Commercial operators, such as major retailers, often hold a lot of data about individuals. They should also have to move towards the empowerment of individuals to say what should or should not be done with data that concerns them.

Defra is almost unique in Whitehall in having vast swathes of data that are non-personal: the majority of Defra data are environmental—covering the land and sea—agricultural, and quite often the result of scientific research to understand diseases in plants and animals. Increasingly, remote sensing and earth observation makes up a large part of those data holdings. Much of those data have traditionally been under-used, and for Defra and other Whitehall Departments tasked with shrinking budgets and an expectation to deliver more in changing circumstances means getting more value from our data was key to meeting that challenge. For non-personal datasets, publishing them as open data allows Defra—which is a complex organisation of over thirty bodies including the core Department, the Environment Agency, Natural England and others—to share data more easily internally as well as externally. It also allows data to be used productively to deliver applications and services by the private, not-for-profit and academic sectors, and also other government departments.

### Defra’s Data Push

In an effort to try and stimulate innovation in the use of data, in 2015 Defra’s Secretary of State set the target of publishing 8,000 open datasets in 12 months^1^ (in the end, more than 10,000 open datasets were published in that timeframe^2^). Much of this concerned an internal challenge to Defra’s staff to start to use data more intelligently, and the first step was to recognise it as an important asset and to make its data as open as possible. Just the process of identifying data sets and publishing them helped to raise the profile of the possibilities. Before this challenge was set, few people thought of Defra as an organisation that is largely founded on data, but that has now changed, even if there is still less than total consensus about the definition of data and its purpose. But Defra, like many other parts of government, is on a journey and clarity will develop in due course.

### Beginning to Build Tools

Defra is now moving out of its engagement phase and starting to build useful tools with its data assets, and this is perhaps where the benefits can be pointed to, rather than relying on rhetoric. There is strong coordination between Defra’s Earth Observation Centre of Excellence, its agencies, and its data and digital transformation programmes, which is beginning to build the digital tools and platform to inform decision making at the policy and operational levels.

**Figure 1:**
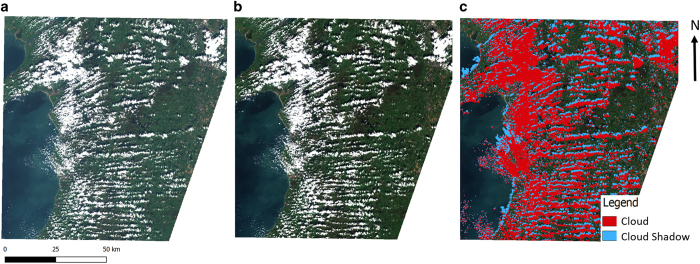
The pre-processing steps visualised from the raw Sentinel-2 data. Available for download from the Copernicus Open Access Hub (**a**); to an analysis ready format of the data that has been corrected for atmospheric conditions and topographic illumination errors (**b**) and cloud masking (**c**). Data were processed by Dr Pete Bunting, Aberystwyth University, using the Atmospheric and Radiometric Correction of Satellite Imagery (ARCSI) open source software package. Website: https://scihub.copernicus.eu/dhus/#/home.

**Figure 2:**
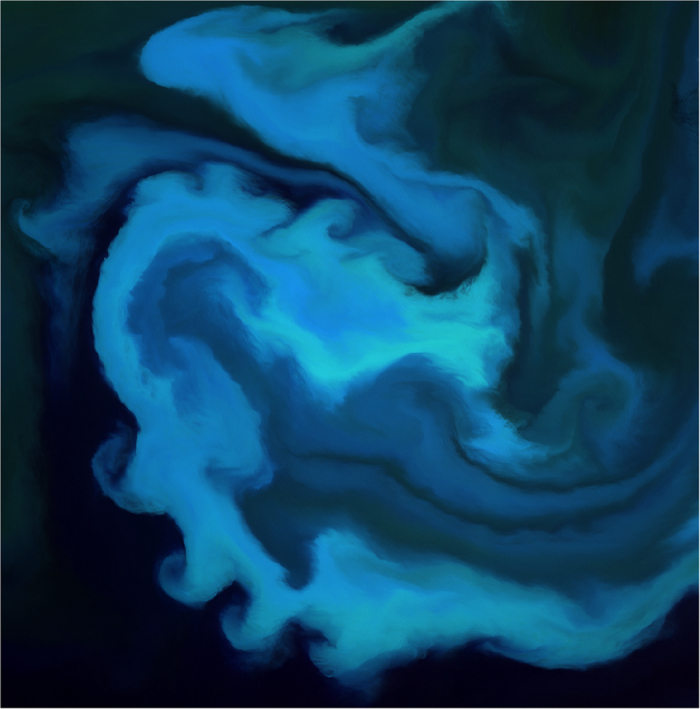
Algal bloom in the South Atlantic Ocean just south of the Falkland Islands captured by Sentinel-2, an optical sensor that captures data at 10 m pixel resolution. Data processed by Dr Gwawr Jones, JNCC.

Some important work concerns fundamentals: for example, making sure that processing needed to correct for atmospheric effects in portions of satellite data captured on different days, and when parts of the Earth’s surface are obscured by cloud cover, is done once centrally and done well (Fig. 1). This ensures that effort is not duplicated or wasted across the Defra group, and indeed across government. Once these fundamentals are in place and we have a clean image with which to work, it’s possible to use machine learning algorithms to detect changes from one day to the next. An area where this is useful is in detecting changes in tree cover in forests, which is something that concerns the Forestry Commission. Storm-damaged forestry estate costs far less to repair days after the storm than weeks after it, which might be the earliest that ground-based inspectors are able to detect a problem. Illegal logging also occurs, so being able to identify logging where it shouldn’t be occurring is important. In both cases, being able to identify a change in the forest in close to real time can help target site visits for rangers and this which is much more cost-effective than spot visits. See another example of how Defra is using satellite date in Fig. 2.

If we can’t learn to use earth observation data for these kinds of applications we’ll struggle with more complex data problems. This is because these are a highly constrained kinds of data problems: the data are clearly defined in terms of source, structure, provenance and continuity, and we know where the data are stored and we have efficient algorithms for access and calibrating the data. But, in general once one tries to move up the information hierarchy it’s very easy to get bogged down. On the face of it, the data are very simple—spectral reflectance for a particular patch of ground, which is accurately referenced in space and time. But allowing people to interact intelligently with these data to extract static and dynamic information is something we haven’t yet cracked properly. Google Maps is about as sophisticated as simple-to-use tools get, but this probably represents just a few percent of the information content and potential of earth observation data. In the hands of specialists it has generated specific useful products that lead to evidence and that drive outcomes, but it’s going to take some smart thinking and new algorithms to crack the problem of making even quite simple data like earth observation useful when in the hands of non-specialists. The challenges with creating useful interfaces between people and much less formal data structures are going to be much greater than for earth observation.

Many of the benefits of building future policies around data are still to be realised. Policies like the EU’s Common Agricultural Policy, which regulates agricultural production and its environmental impact through a system of quotas and subsidies, are very difficult to implement because they pre-date data that can be easily obtained and stored. In future, the UK leaving the EU, while not without challenges, provides an opportunity to design policies that are founded on reliable data flows so that they can be implemented much more effectively.

## Additional information

**How to cite this article:** Boyd, I. L. The stuff and nonsense of open data in government. *Sci. Data* 4:170131 doi: 10.1038/sdata.2017.131 (2017).
